# Epigenetic modification of BDNF mediates neuropathic pain via miR-30a-3p/EP300 axis in CCI rats

**DOI:** 10.1042/BSR20194442

**Published:** 2020-11-13

**Authors:** Ming Tan, Lulu Shen, Yayun Hou

**Affiliations:** 1 Department of Anesthesiology, The Central Hospital of Enshi Tujia And Miao Autonomous Prefecture, Enshi Clinical College of Wuhan University, Enshi, Hubei, China; 2 Department of Anesthesiology, Huai’an Second People’s Hospital and The Affiliated Huai’an Hospital of Xuzhou Medical University, No. 66 Huaihai South Road, Huai’an, Jiangsu, China; 3 Department of Anesthesiology, Huai’an Hospital of Traditional Chinese Medicine, Heping Road, Huai’an, Jiangsu, China

**Keywords:** BDNF, EP300, Epigenetic modification, miR-30a-3p, Neuropathic pain

## Abstract

Recent investigation of microRNAs on chronic pain has developed a breakthrough in neuropathic pain management. In the present study, decreased expression of miR-30a-3p was reported using qRT-PCR analysis and loss of miR-30a-3p promoted neuropathic pain progression in sciatic nerve chronic constrictive injury rats through determining the pain threshold. We predicted miR-30a-3p could target E-cadherin transcriptional activator (EP300) via bioinformatics analysis. Meanwhile, we found that brain-derived neurotrophic factor (BDNF) is involved in neuropathic pain. Here, we exhibited that EP300 epigenetically up-regulated BDNF via enhancing acetylated histone H3 and H4 on the promoter. For another, miR-30a-3p was able to modify the level of BDNF and acetylated histone H3 and H4. Loss of miR-30a-3p enhanced EP300 and BDNF colocalization in CCI rats. Subsequently, it was shown that increased EP300 induced neuropathic pain by an enhancement of neuronal BDNF level *in vivo*. To sum up, it was revealed that epigenetic modification of BDNF promoted neuropathic pain via EP300 induced by miR-30a-3p in CCI rats.

## Introduction

Neuropathic pain, which can develop after the nerve injury, is a common chronic pain. Twenty percent cancer pains, which result from chemotherapy side effects, are due to the origin of neuropathic pain [1,2]. Nowadays, its prevalence still is very high and the treatments still exhibit disappointing efficacy [3]. Although decades have been spent to identify its specific mechanisms, further investigation is still needed.

MicroRNAs can play a significant role in modulation of genes [4,5]. Via binding with the targeting mRNAs, microRNAs can post-transcriptionally regulate genes [6]. Altered expression of microRNAs in nerve system has been implicated in neuropathic pain [7–9]. miR-93 reduces the development of neuropathic pain through targeting STAT3 [10]. Down-regulation of miR-221 represses neuropathic pain development via regulating SOCS1 [11]. In addition, loss of miR-155 greatly attenuates neuropathic pain progression [12].

miR-30 is highly conserved and there are five family members. miR-30 can exhibit different roles and they function in various cancers. Inhibition of miR-30a can induce ESCC proliferation via activating Wnt signaling [13]. Wnt pathway is known to be involved in neuropathic pain [14]. In addition, the biological roles of miR-30 have been implicated in neuropathic pain. For example, miR-30b is decreased in neuropathic pain in rat models. miR-30a-5p in the serum of rats after SNL surgery was greatly down-regulated [15].

Mature brain-derived neurotrophic factor (mBDNF) has been reported to exert a vital role in the nervous system [16]. Primary afferent-derived BDNF can contribute to the development of pain [17]. In addition, sortilin gates neurotensin and BDNF signaling to regulate peripheral neuropathic pain [18]. Previously, miR-30a-5p can repress BDNF expression in prefrontal cortex [19]. E-cadherin transcriptional activator (EP300) is identified as a transcriptional co-activator and it was predicted as a downstream target of miR-30a-5p using bioinformatics tools. Knockdown of EP300 in L6-S2 DRG neurons of rats reduced histone acetylation and repressed chronic stress-induced visceral pain [20]. Nevertheless, the mechanism of miR-30a-3p, EP300 and BDNF in the progression of neuropathic pain is yet to be studied.

We found that loss of miR-30a-3p induced neuropathic pain through increasing EP300 level, which could epigenetically modify BDNF. We aimed to investigate the detailed role of miR-30a-3p in neuropathic pain and its underlying mechanism.

## Materials and methods

### Neuropathic pain model

Fifty-six SD rats (aged 6–8 weeks and weighing 180–200 g) were purchased from Shanghai Animal Laboratory Center. We carried out the animal experiments in a climate-controlled Animal Center in Xuzhou Medical University with a 12-h light/dark cycle. Food and water *ad libitum* were well provided. CCI rat model was conducted based on a previous method [21]. Rats were anesthetized using 40 mg/kg sodium pentobarbital. Then, on both sides of the rats, a mid-thigh incision was used to expose the sciatic nerves. We used a 4-0 catgut thread to ligate the sciatic nerves at four sites. Sciatic nerve was exposed and isolated without ligation was employed as the sham control group. Animal protocols were based on the requirements of Institutional Animal Care. Animal handling was carried out based on the policies of the Guide for Care and Use of Laboratory Animals and was approved by the Ethical Committee for Animal Experimentation of Huai’an Second People’s Hospital. Every effort was done to relieve their stress. The rats were killed using intravenous administration of 140 mg/kg pentobarbital.

### Intrathecal injections

We separated the occipital muscles and inserted PE-10 polyethylene catheter into the cisterna magna. Then, intrathecal implantation was validated using bilateral hindlimbs paralysis injected using lidocaine. Two days later, CCI was carried out. Three days before the surgery, to deliver the genes, 10 μl recombinant lentivirus, LV-EP300, LV-shEP300, miR-30a-3p inhibitors or mimics (GenePharma, Shanghai, China) were injected. Afterward, the rats were classified into the following groups at random: (1) sham group; (2) CCI model group; (3) CCI + LV-NC group; (4) CCI + LV-shEP300; (5) CCI + LV-EP300; (6) CCI + miR-30a-3p mimics; (7) CCI + miR-30a-3p inhibitors. We diluted the lentiviruses using 0.2 ml complete medium at 10^7^ transduction units/ml added with hexadimethrine bromide (Polybrene; 8 mg/ml). Eight mice were used in each group. After surgery, rats were killed to isolate L4–L6 dorsal spinal cords at days 0, 3, 7, 14 and 21.

### Determination of pain threshold

Mechanical allodynia was detected using PWT, which can respond to Von Frey filaments [22]. Before test, to adapt to the environment, rats were left for half an hour. The plantar surface of hind paw was given pressure using the electronic Von Frey filament. We recorded the force at the time of paw withdrawal. Then, PWL was evaluated using heat sensitivity responding to radiant heat using the Hargreaves method [23]. On an elevated glass table, rats were maintained in perspex boxes. A radiant heat source was applied on the center of the plantar surface of hind paws under glass table. To induce PWL, we set the heat intensity at 10 s with 20 s cut-off time. An interval of 5–10 min was used to give heat stimulus.

### Cell culture

Rat microglia cells were purchased from Sciencell (Carlsbad, CA, U.S.A.). Microglia medium was used to incubate the rat microglia cells with 5% CO_2_ at 37°C.

### qRT-PCR

We extracted total RNA using TRIzol (Invitrogen, Carlsbad, CA, U.S.A.). To extract small RNAs, miRVana kits (Ambion Inc., Austin, TX, U.S.A.) were used. Then, cDNA was reverse-transcribed using M-MLV reverse transcriptase (Clontech, Palo Alto, CA, U.S.A.). SYBR Green qPCR Master Mix (Takara, Dalian, China) was used. The primer sequences are exhibited in Table 1. The 2^−ΔΔ*C*_t_^ was used to quantify relative gene expression.

**Table 1:** Primers used for real-time PCR

Genes	Forward (5′–3′)	Reverse (5′–3′)
*GAPDH*	CAAGGTCATCCATGACAACTTTG	GTCCACCACCCTGTTGCTGTAG
*U6*	CTCGCTTCGGCAGCACA	AACGCTTCACGAATTTGCG
*EP300*	AAAAATAAGAGCAGCCTGAG	AGACCTCTTTATGCTTCTTCC
*BDNF*	GTAGTTTTTGTAGGATGAGGAAGTG	TATAAATTAACAACCCCAATACACA
*miR-30a-3p*	CGCTTTCAGTCGGATGTTTG	GTGCAGGGTCCGAGGT

### Bioinformatics analysis

TargetScan (http://www.targetscan.org/), Starbase (http://starbase.sysu.edu.cn/), miRanda (http://www.microrna.org/) and miRDB (http://www.mirdb.org/) databases were used to predict EP300 as a putative target of miR-30a-3p.

### Dual-luciferase reporter assay

3′-UTR of EP300 with the sequences of the miR-30a-3p, WT or MUT 3′-UTR of EP-300 were cloned into pmirGLO dual-luciferase vector. Afterward, the vectors were co-transfected with miR-30a-3p mimics. Cells were collected and luciferase activity was measured using the Dual-Luciferase Assay System (Promega, Madison, WI, U.S.A.).

### Western blot

Proteins were loaded on 10% sodium dodecyl sulfate polyacrylamide gels. Then, PVDF membrane was utilized. Membranes were treated with 5% non-fat dry milk. Primary antibodies were used at 4°C for a whole night. After rinsing using TBST, the second antibodies included: HRP-Conjugated AffiniPure Goat Anti-Rabbit IgG (1:2000; Beyotime, Shanghai, China) and HRP-Conjugated AffiniPure Goat Anti-Mouse IgG (1:2000; Beyotime, Shanghai, China). The bands were visualized using Pierce ECL Western Blotting Substrate (Pierce, Rockford, IL, U.S.A.). Primary antibodies included anti-EP300 (1:1000), BDNF (1:1000) and anti-GAPDH (1:1000) (Cell Signaling Technology, Danvers, MA, U.S.A.).

### Immunohistochemical staining

Spinal dorsal horn of the rats was perfused using 4% paraformaldehyde and 2.1% picric acid for half an hour. Then, spinal cords were fixed at 4°C for a whole night. A thickness of 20 μm coronal sections were cut using a microslicer. Primary antibodies against EP300, BDNF, NeuN (Cell Signaling Technology, Danvers, MA, U.S.A.) and a fluorescent-conjugated secondary antibody were used. A laser confocal microscope (LSM710; Carl Zeiss, Germany) was used to obtain fluorescence images.

### ChIP assay

ChIP assays were carried out based on the protocol from Millipore (Billerica, MA, U.S.A.). Cells were cross-linked using 1% formaldehyde and re-suspended using lysis buffer and then sonicated on wet ice. Afterward, immuno-clearing was carried out with 45 μl of protein-A-Sepharose/sheared salmon sperm DNA. Samples were immunoprecipitated using acetylated histone H3 or H4 antibody or a non-specific antibody, normal rabbit immunoglobulin. Finally, we analyzed the products using primers for a region on the BNDF promoter.

### Statistical analysis

Results were analyzed by SPSS 19.0. Then, statistical significance between two groups was analyzed using Student’s *t* test. To determine the statistical significance among multiple groups, one-way analysis of variance was conducted. Differences were considered significant with *P*-value <0.05.

## Results

### Decrease in miR-30a-3p contributed to neuropathic pain in CCI rat models

**Figure 1:**
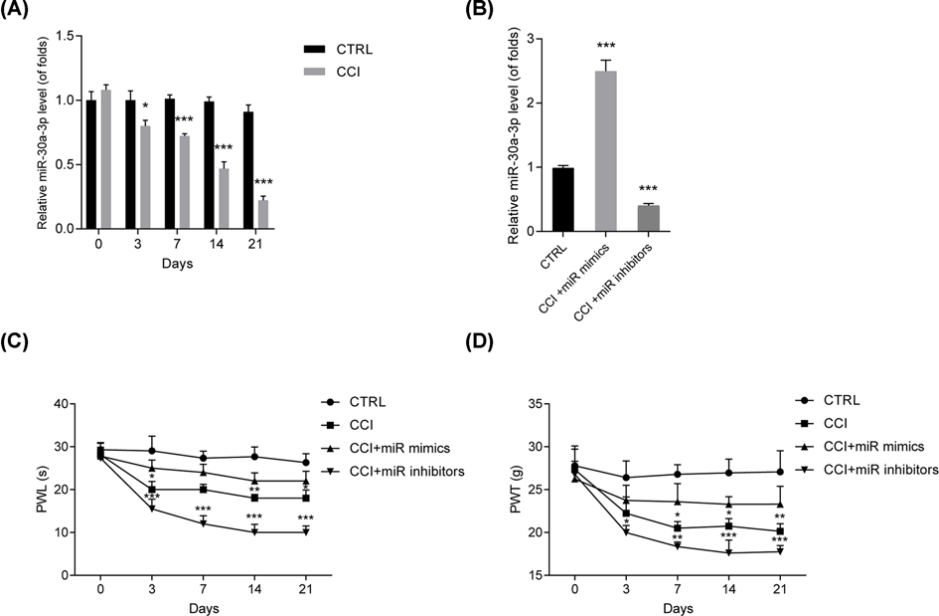
Decreased level of miR-30a-3p contributed to the development of neuropathic pain in CCI model rats (**A**) miR-30a-3p decreased in the spinal cord in CCI rats. (**B**) Levels of miR-30a-3p after microinjection of miR-30a-3p mimics and inhibitors. (**C,D**) Effects of miR-30a-3p on PWL and PWT. Behavior was tested at 0, 3, 7, 14 and 21 days after CCI. *n*=8 for each group, three independent experiments were carried out. Error bars stand for the mean ± SD of at least triplicate experiments. *P<0.05, **P<0.01, ***P<0.001.

First, to study the function of miR-30a-3p in neuropathic pain, RT-qPCR was carried out. miR-30a-3p was obviously decreased in CCI rats compared with the levels in sham group at postoperative days 3, 7, 14 and 21 as shown in Figure 1A **(*P*<0.001)**. Then, the levels of miR-30a-3p were modulated using microinjection of miR-30a-3p mimics and inhibitors in CCI rats to study the detailed effect of miR-30a-3p on neuropathic pain. In Figure 1B, 2 weeks later, we found that miR-30a-3p mimics greatly elevated miR-30a-3p expression while it was successfully inhibited by the inhibitors **(*P*<0.001)**. Then, effects of miR-30a-3p on PWL and PWT were assessed. We proved that silence of miR-30a-3p repressed PWL (Figure 1C) and PWT (Figure 1D) **(*P*<0.05)**, while miR-30a-3p inhibitors increased that. These indicated miR-30a-3p could relieve neuropathic pain development.

### EP300 was a target of miR-30a-3p and depressed by miR-30a-3p

**Figure 2:**
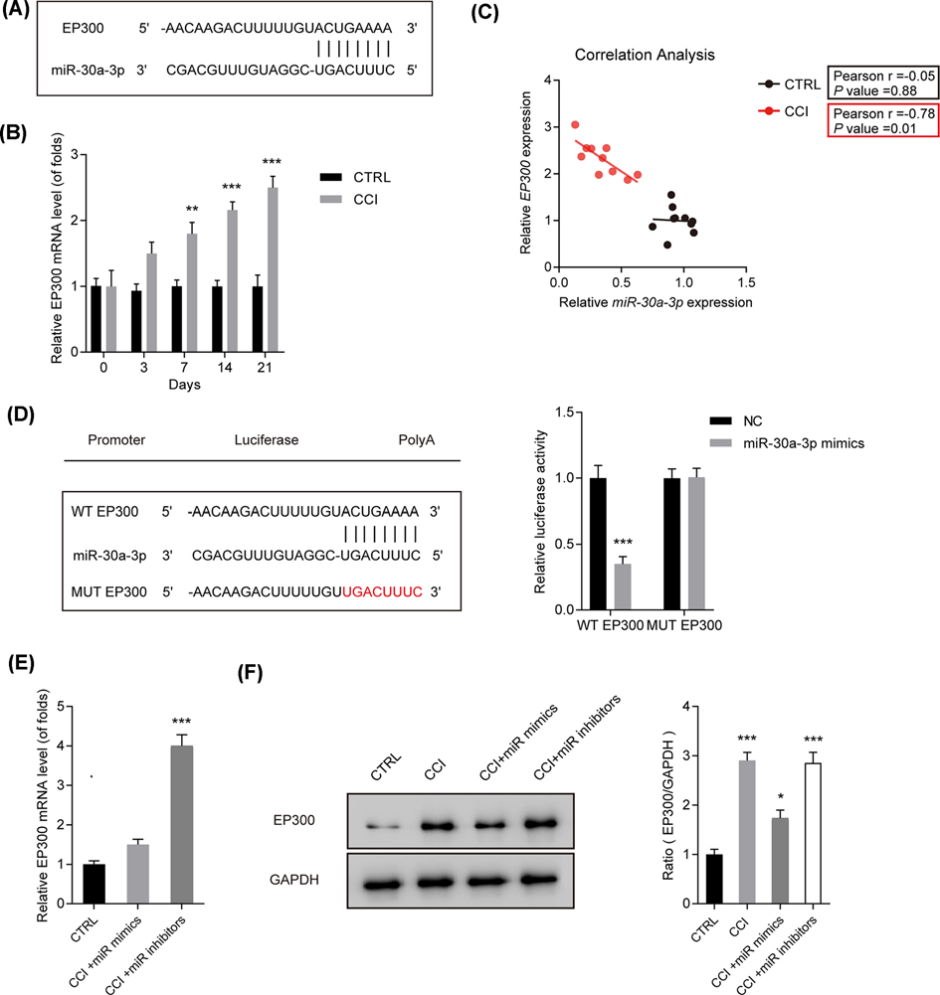
EP300 was a target of miR-30a-3p and was down-regulated by miR-30a-3p (**A**) Potential binding sites of EP300 and miR-30a-3p. (**B**) Expression of EP300 mRNA in the spinal cord after CCI. (**C**) Correlations between the expressions of EP300 and miR-30a-3p *in vivo*, calculated by Spearman coefficients. (**D**) The dual-luciferase reporter assay of EP300 and miR-30a-3p. (**E,F**) mRNA and protein levels of EP300 detected by q-PCR and Western blot. CCI rats were given microinjection of miR-30a-3p mimics and inhibitors. Three independent experiments were carried out. Error bars stand for the mean ± SD of at least triplicate experiments. *P<0.05, **P<0.01, ***P<0.001.

Next, EP300 was predicted as a target for miR-30a-3p via using bioinformatics analysis. Their potential binding sites were exhibited in Figure 2A. In Figure 2B, expression of EP300 mRNA in the spinal cord after CCI was induced in a time-dependent course **(*P*<0.01)**. For another, correlation between the expressions of EP300 and miR-30a-3p *in vivo*, was calculated using Spearman coefficients. As exhibited in Figure 2C, a reverse association between EP300 and miR-30a-3p was observed in CCI rat models after 14 days **(*P*=0.01)**. Subsequently, we carried out the dual-luciferase reporter assay to confirm the correlation of EP300 and miR-30a-3p (Figure 2D). Co-transfection of WT-EP300 and miR-30a-3p mimics strongly suppressed the reporter activity in rat microglial cells (Figure 2D) **(*P*<0.001)**. As evidenced in Figure 2E,F **(*P*<0.05)**, EP300 expression was modulated by miR-30a-3p negatively *in vivo*. These indicated EP300 was a target of miR-30a-3p.

### EP300 epigenetically up-regulated BDNF via enhancing acetylated histone H3 and H4 on its promoter

**Figure 3:**
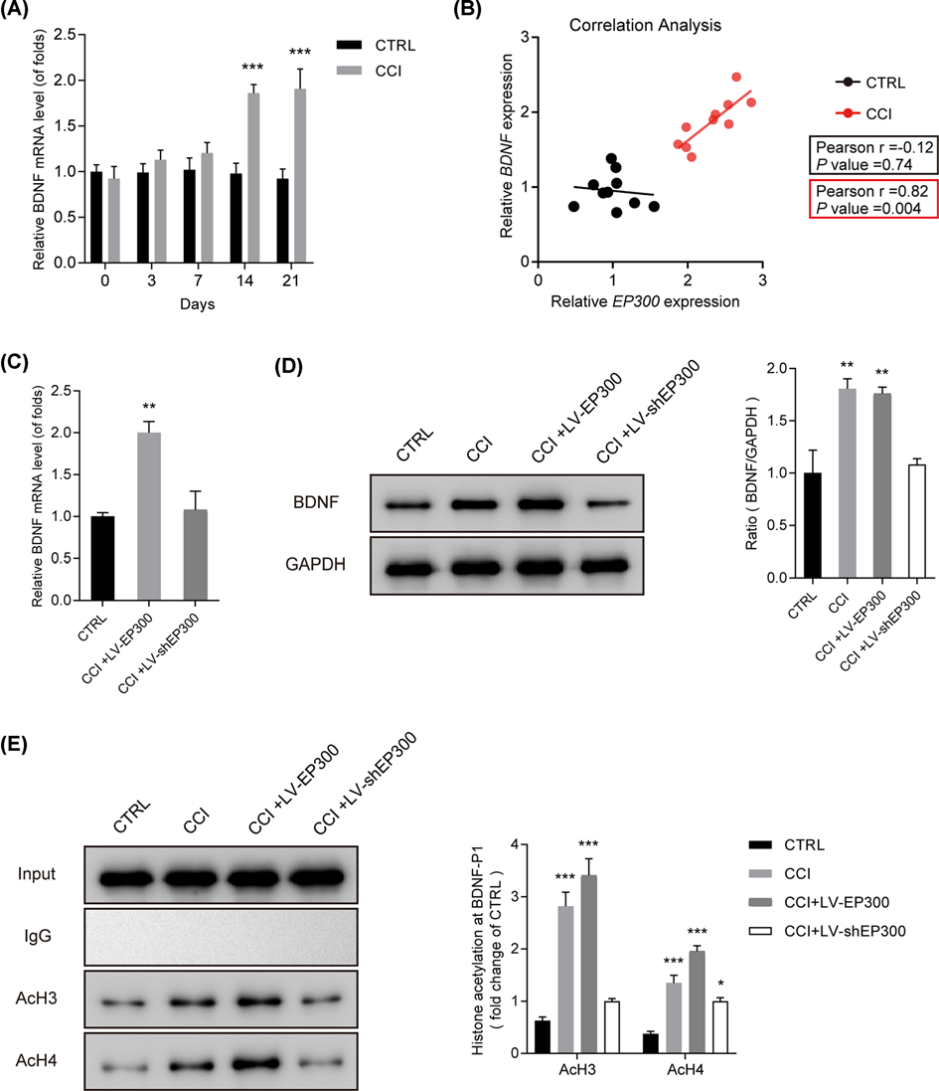
EP300 epigenetic up-regulated BDNF via enhancing the level of acetylated histone H3 and H4 on BDNF promoter (**A**) BDNF increased in the spinal cord in CCI rats. (**B**) Correlations between the expressions of EP300 and BDNF *in vivo*, calculated by Spearman coefficient. (**C,D**) mRNA and protein levels of BDNF detected by q-PCR and Western blot. CCI rats were given microinjection of LV-EP300 and LV-shEP300. (**E**) Levels of AcH3 and AcH4 binding to the BDNF promoter detected by ChIP assay. CCI rats were given microinjection of LV-EP300 and LV-shEP300. Three independent experiments were carried out. Error bars stand for the mean ± SD of at least triplicate experiments. *P<0.05, **P<0.01, ***P<0.001.

Moreover, BDNF was increased in CCI rats especially at postoperative days 14 and 21 (Figure 3A) **(*P*<0.001)**. Correlation between the expressions of EP300 and BDNF *in vivo* was measured using Spearman coefficients. A positive correlation between EP300 and BDNF was manifested in Figure 3B **(*P*=0.004).** Meanwhile, CCI rats were microinjected by LV-EP300 and LV-shEP300. We observed that BDNF expression was promoted by LV-EP300 as exhibited in Figure 3C,D **(*P*<0.01).** Then, levels of AcH3 and AcH4 binding to the BDNF promoter were detected using ChIP assay. It was displayed that Figure 3E **(*P*<0.05)**, EP300 enhanced the level of acetylated histone H3 and H4 on BDNF promoter. It was indicated that EP300 epigenetically activated BDNF via enhancing acetylated histone H3 and H4 levels on BDNF promoter.

### miR-30a-3p repressed the level of BDNF via inactivating the level of acetylated histone H3 and H4 on BDNF promoter

**Figure 4:**
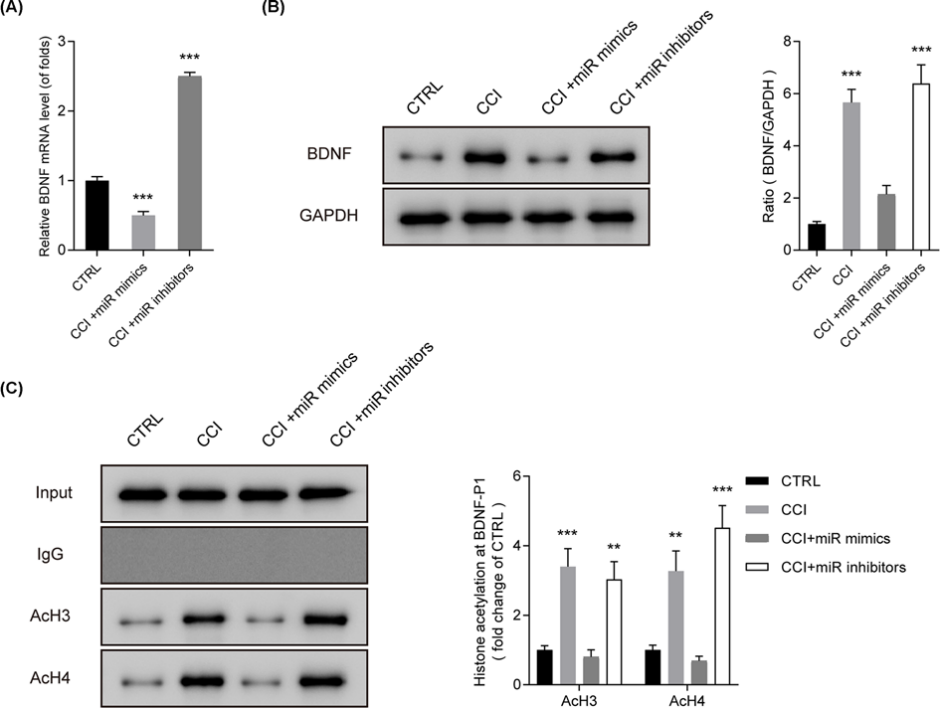
miR-30a-3p modified the level of BDNF and acetylated histone H3 and H4 on BDNF promoter (**A,B**) mRNA and protein levels of BDNF detected by q-PCR and Western blot. CCI rats were microinjection of miR-30a-3p mimics and inhibitors. (**C**) Levels of AcH3 and AcH4 binding to the BDNF promoter detected by ChIP assay. CCI rats were given microinjection of miR-30a-3p mimics and inhibitors. Three independent experiments were carried out. Error bars stand for the mean ± SD of at least triplicate experiments. **P<0.01, ***P<0.001.

Furthermore, as displayed in Figure 4A,B **(*P*<0.001)**, expression of BDNF was negatively regulated by miR-30a-3p. ChIP assay was utilized to determine AcH3 and AcH4 levels binding to the BDNF promoter. In Figure 4C **(*P*<0.01)**, miR-30a-3p inactivated the level of acetylated histone H3 and H4. These data implied that miR-30a-3p might inhibit BDNF via inactivating acetylated histone H3 and H4 on its promoter.

### Down-regulation of miR-30a-3p induced colocalization of EP300 and BDNF

**Figure 5:**
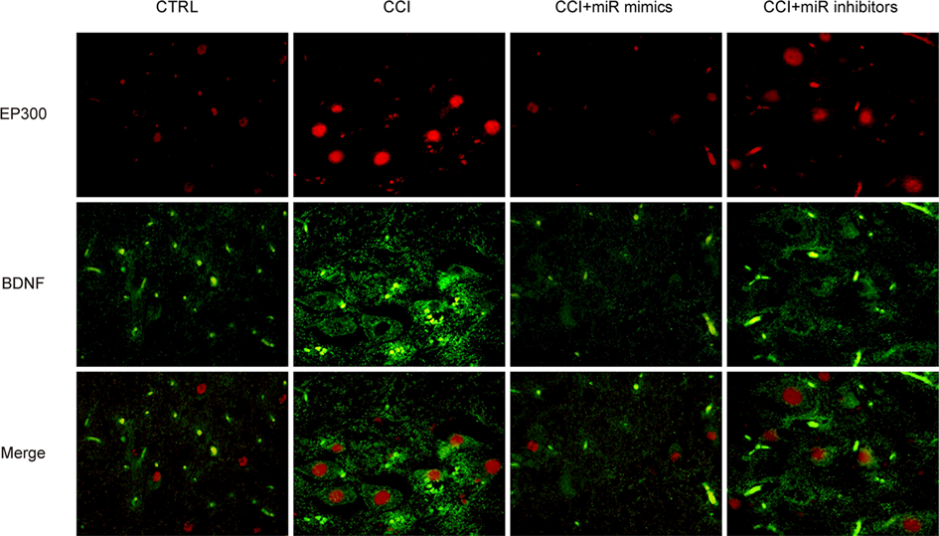
Inhibition of miR-30a-3p increased the colocalization of EP300 and BDNF in the spinal dorsal horn of CCI rats Double immunofluorescence staining of EP300 and BDNF in the spinal dorsal horn of CCI rats. CCI rats were given microinjection of miR-30a-3p mimics and inhibitors. Three independent experiments were carried out. Error bars stand for the mean ± SD of at least triplicate experiments.

Then, double immunofluorescence staining of EP300 and BDNF in CCI rats was performed. As exhibited in Figure 5, in the spinal dorsal horn, loss of miR-30a-3p induced the colocalization of EP300 and BDNF. These indicated the colocalization of EP300 and BDNF in CCI rats was negatively modulated by miR-30a-3p.

### EP300 promoted neuropathic pain through up-regulating neuronal BDNF in the spinal dorsal horn of CCI rats

**Figure 6:**
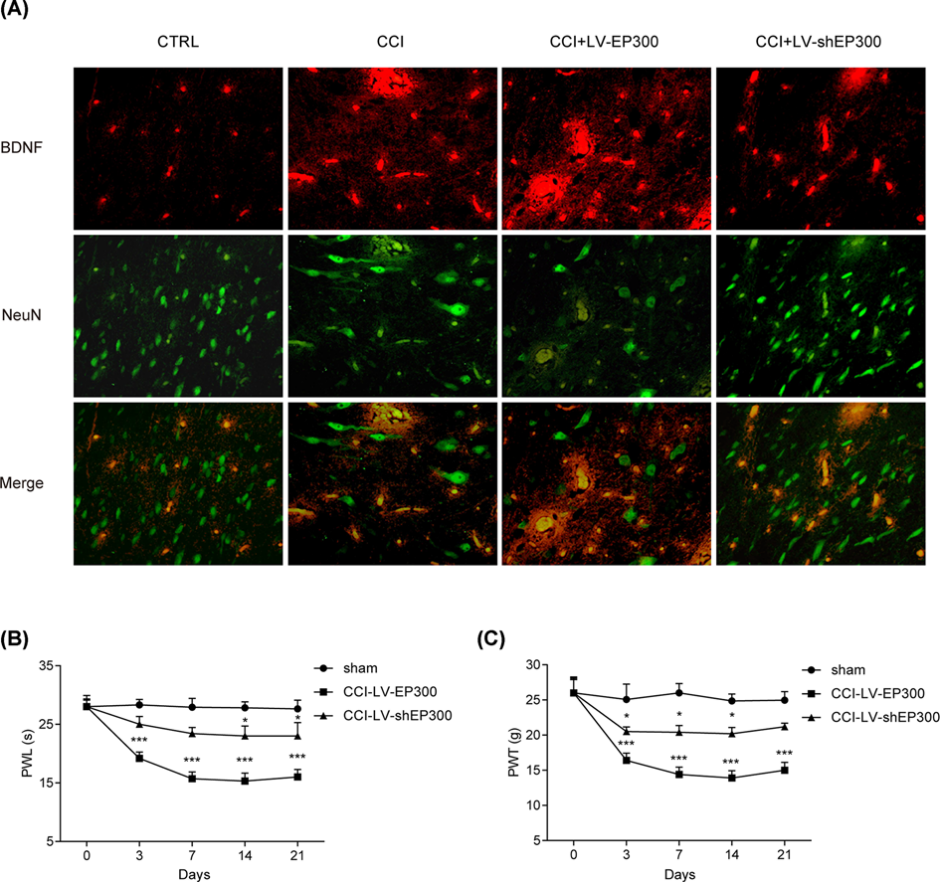
Increased EP300 promoted neuropathic pain by up-regulating the level of neuronal BDNF in the spinal dorsal horn of CCI rats (**A**) Double immunofluorescence staining of NeuN (neuronal marker) and BDNF in the spinal dorsal horn of CCI rats. CCI rats were given microinjection of LV-EP300 and LV-shEP300. (**B,C**) Effects of EP300 on PWL and PWT. *n*=8 for each group, three independent experiments were carried out. Error bars stand for the mean ± SD of at least triplicate experiments. *P<0.05, ***P<0.001.

Next, double immunofluorescence staining of NeuN (neuronal marker) and neuronal BDNF in CCI rats was carried out. In Figure 6A, immunofluorescence staining indicated that BDNF in the spinal dorsal horn was induced by EP300, while reduced by loss of EP300. PWL and PWT were obviously decreased by loss of EP300 and overexpression of EP300 accelerated neuropathic pain in Figure 6B,C **(*P*<0.05)**. These indicated that EP300 induced neuropathic pain through up-regulating neuronal BDNF in the spinal dorsal horn of CCI rats.

## Discussion

Recently, in neuropathic pain, increasing microRNAs were demonstrated [7,24]. Currently, we reported miR-30a-3p was decreased in neuropathic pain. Loss of miR-30a-3p greatly induced neuropathic pain in CCI rats. EP300 was predicted as the target of miR-30a-3p, which could be negatively modulated by miR-30a-3p. In addition, we reported that EP300 could epigenetically activate BDNF via enhancing acetylated histone H3 and H4 expression on BDNF promoter. Meanwhile, miR-30a-3p modified the acetylated histone H3 and H4 on BDNF promoter. Knockdown of miR-30a-3p elevated the colocalization of EP300 and BDNF in CCI rats, whereas its overexpression reduced that. EP300 obviously contributed to the neuropathic pain through enhancing BDNF level.

Increasing studies have studied the role of miR-30a, which is widely known to participate in many cellular processes [25]. For instance, miR-30a represses glioma progression via repressing Wnt5a [26]. miR-30a-5p can ameliorate inflammation and oxidative stress triggered by spinal cord injury through targeting Neurod 1 and MAPK/ERK [27]. Currently, we found miR-30a-3p was obviously down-regulated in CCI rat models. In a mouse model of traumatic peripheral nerve injury, a persistent intimal hypoxia can cause neuralgia with elevated levels of HIF-1α [28]. HIF-1α and HIF-2α can regulate miR-30a-3p expression negatively [29]. Then, we found loss of miR-30a-3p contributed to the progression of neuropathic pain. Then, we found EP300 was a potential target gene of miR-30a-3p. EP300 has been originally recognized as a transcriptional co-activator, which can play pivotal roles in transcription events [30]. The most well-studied effect of EP300 is that it can act as a histone acetyltransferase, which can regulate transcription through chromatin remodeling [31]. In addition, it has significant roles in multiple biological processes [32]. Here, in our present data, we implied that miR-30a-3p modulated EP300 expression negatively.

Neurotrophins such as BDNF are extensively studied in neuropathic pain progression [33]. BDNF signaling can demonstrate a well-reported function in psychiatric disorders [34]. BDNF can drive neuropathic pain progression [35]. Previously, it has been shown that CCI elevates BDNF expression, which contributes a lot to the nociception [36]. BDNF from microglia can lead to the shift in neuronal anion gradient [37]. BDNF-activated JNK induces the progression of neuropathic pain [38]. Meanwhile, miR-206 can ameliorate CCI-triggered neuropathic pain through MEK/ERK signaling via targeting BDNF [39]. In addition, BDNF induces neuropathic pain via activating SHP2 correlated GluN2B-containing NMDA receptors in SNL rat models [40].

Next, it has been reported that histone acetylation can regulate BDNF transcription [41,42]. Here, we reported that EP300 epigenetically up-regulated BDNF levels via inducing the level of acetylated histone H3 and H4. miR-30a-3p could modify the level of BDNF via targeting the acetylated histone H3 and H4 on BDNF promoter. The colocalization of EP300 and BDNF in the spinal dorsal horn was increased by loss of miR-30a-3p. In addition, the influence of miR-30a-3p on BDNF is indirect since BDNF is not the direct target for miR-30a-3p based on the bioinformatics analysis. It was suggested in our study that miR-30a-3p might inhibit BDNF via inactivating acetylated histone H3 and H4 on its promoter. More signaling pathway regulating BDNF should also be included to strengthen our data. We found that miR-30a-5p, EP300 and BDNF present different expressions along time after CCI surgery. In our future study, we would like to investigate this chronological difference, which might involve other signaling pathway.

To sum up, a crucial function of miR-30a-3p in neuropathic pain progression was indicated. Decreased miR-30a-3p induced neuropathic pain in CCI model rats. We manifested that miR-30a-3p might involve the EP300-mediated BDNF activation via targeting the acetylated histone H3 and H4 on BDNF promoter in neuropathic pain. Our study suggested that epigenetic modification of BDNF promoted neuropathic pain via EP300 induced by miR-30a-3p in CCI rats.

## Data Availability

All data are available upon request.

## Abbreviations

BDNF: brain-derived neurotrophic factor
CCI: chronic constriction injury
ChIP: Chromatin immunoprecipitation
EP300: E-cadherin transcriptional activator
ESCC: esophageal squamouscell carcinoma
GluN2B: glutamate ionotropic receptor NMDA type subunit 2B
HIF-1: hypoxia-inducible factor-1
NMDA: N-methyl-D-aspartic acid receptor
PWL: paw withdrawal latency
PWT: paw withdrawal thresholds
RT-qPCR: Real-time quantitative Polymerase Chain Reaction
SD rat: Sprague Dawley rat
SHP2: Src homology region 2-containing protein tyrosine phosphatase 2
SNL: spinal nerve ligation
SOCS: Suppressor of cytokine signalling
